# REGIONAL POLICY SCAN: POLICIES TO ADDRESS AGING IN SUB-SAHARAN AFRICA

**DOI:** 10.1093/geroni/igae098.2780

**Published:** 2024-12-31

**Authors:** Edward Osae-Oppong

**Affiliations:** California State University San Marcos, San Marcos, California, United States

## Abstract

This paper presenting a regional policy scan focused on aging in sub-Saharan Africa. The study aims to provide an overview of policy responses to aging in the region, identifying key challenges, opportunities, and recommendations for addressing the needs of the aging population. The methodology involves conducting a critical essay reviewing articles on public policy related to mental health and well-being in older populations across databases such as PubMed, Google Scholar, and Science Direct. This review analyzes policies addressing healthcare needs and identifies demographic, economic, and social trends to understand the aging population comprehensively. Findings from the policy scan reveal a growing aging population in sub-Saharan Africa, with existing policies and strategies in place to address their needs. However, challenges persist in implementing effective policies, social protection systems, and engaging the private sector. Key recommendations include advocating for a comprehensive and coordinated approach to aging, improving access to healthcare and social services, incentivizing employers to hire older workers, and increasing public awareness of aging issues. The study concludes that sub-Saharan Africa faces significant challenges and opportunities concerning its aging population. It underscores the importance of developing and implementing effective policies and strategies to address the healthcare needs of older populations, strengthen social protection systems, and engage the private sector. By investing in the health and well-being of older adults, sub-Saharan Africa can achieve sustainable economic development and enhance the quality of life for its aging population.

